# Clinical, genetic, and functional characterization of the glycine receptor β-subunit A455P variant in a family affected by hyperekplexia syndrome

**DOI:** 10.1016/j.jbc.2022.102018

**Published:** 2022-05-06

**Authors:** Ghada I. Aboheimed, Maha M. AlRasheed, Sultan Almudimeegh, Karla A. Peña-Guerra, Kelly J. Cardona-Londoño, Mustafa A. Salih, Mohammed Z. Seidahmed, Futwan Al-Mohanna, Dilek Colak, Robert J. Harvey, Kirsten Harvey, Stefan T. Arold, Namik Kaya, Arnaud J. Ruiz

**Affiliations:** 1 Department of Translational Genomics, Center for Genomic Medicine, King Faisal Specialist Hospital and Research Centre, Riyadh, Kingdom of Saudi Arabia; 2 Department of Clinical Pharmacy, College of Pharmacy, King Saud University, Riyadh, Kingdom of Saudi Arabia; 3 Department of Pharmacology, The School of Pharmacy, University College London, London, United Kingdom; 4 Department of Pharmacology and Toxicology, College of Pharmacy, King Saud University, Riyadh, Kingdom of Saudi Arabia; 5 Computational Bioscience Research Center, King Abdullah University of Science and Technology, Thuwal, Kingdom of Saudi Arabia; 6 Department of Pediatrics, College of Medicine, King Saud University, Riyadh, Kingdom of Saudi Arabia; 7 Department of Pediatrics, Security Forces Hospital, Riyadh, Kingdom of Saudi Arabia; 8 Department of Cell Biology, King Faisal Specialist Hospital and Research Centre, Riyadh, Kingdom of Saudi Arabia; 9 Department of Molecular Oncology, King Faisal Specialist Hospital and Research Centre, Riyadh, Kingdom of Saudi Arabia; 10 School of Health and Behavioural Sciences, University of the Sunshine Coast, Maroochydore, Queensland, Australia; 11 Sunshine Coast Health Institute, Birtinya, Queensland, Australia; 12 Centre de Biologie Structurale, CNRS, INSERM, Université de Montpellier, Montpellier, France

**Keywords:** autozygosity mapping, coimmunoprecipitation, confocal imaging, exome sequencing, *GLRB*, immunohistochemistry, patch clamp, startle disease, GlyR, glycine receptor, NGS, next-generation sequencing, TM, transmembrane

## Abstract

Hyperekplexia is a rare neurological disorder characterized by exaggerated startle responses affecting newborns with the hallmark characteristics of hypertonia, apnea, and noise or touch-induced nonepileptic seizures. The genetic causes of the disease can vary, and several associated genes and mutations have been reported to affect glycine receptors (GlyRs); however, the mechanistic links between GlyRs and hyperekplexia are not yet understood. Here, we describe a patient with hyperekplexia from a consanguineous family. Extensive genetic screening using exome sequencing coupled with autozygome analysis and iterative filtering supplemented by *in silico* prediction identified that the patient carries the homozygous missense mutation A455P in *GLRB*, which encodes the GlyR β-subunit. To unravel the physiological and molecular effects of A455P on GlyRs, we used electrophysiology in a heterologous system as well as immunocytochemistry, confocal microscopy, and cellular biochemistry. We found a reduction in glycine-evoked currents in N2A cells expressing the mutation compared to WT cells. Western blot analysis also revealed a reduced amount of GlyR β protein both in cell lysates and isolated membrane fractions. In line with the above observations, coimmunoprecipitation assays suggested that the GlyR α_1_-subunit retained coassembly with β^A455P^ to form membrane-bound heteromeric receptors. Finally, structural modeling showed that the A455P mutation affected the interaction between the GlyR β-subunit transmembrane domain 4 and the other helices of the subunit. Taken together, our study identifies and validates a novel loss-of-function mutation in GlyRs whose pathogenicity is likely to cause hyperekplexia in the affected individual.

Hyperekplexia, also known as hereditary startle disease, is a neurological disorder characterized by neonatal hypertonia and exaggerated startle response to acoustic or tactile stimuli (1, 2). Although rare, hyperekplexia can have serious complications such as injurious falls, brain damage, or sudden infant death. Initial reports suggested that the disease is predominantly an autosomal dominant disorder (3, 4). However, recent studies indicate that recessive cases are more common (5, 6, 7, 8). By far, the most well-studied proteins linked to hyperekplexia belong to the glycine receptor (GlyR) family (9, 10, 11). Human GlyRs form heteropentameric glycine-gated ion channels composed of four ligand-binding α-subunits assembled with one β-subunit (12, 13). The five subunits are arranged pseudosymmetrically around the ion-conducting pore (14). Each subunit consists of three functional domains which are as follows: a transmembrane (TM) domain, an extracellular NH_2_ domain, and a large intracellular domain. The TM domain consists of four amphipathic alpha helices (TM1-TM4). Their main role is to surround the ion channel and form a barrier against the apolar region of the lipid bilayer. Heteromeric GlyRs mediate fast inhibitory neurotransmission mostly in the brainstem and spinal cord and are clustered at postsynaptic sites at glycinergic synapses. The disease is mainly caused by mutations in *GLRA1* encoding the GlyR α_1_ subunit (4, 6, 15, 16, 17, 18, 19, 20) or mutations in *SLC6A5* encoding the glycine transporter type-2 GlyT2 (5). However, deleterious variants in other protein-encoding genes have been linked to hyperekplexia, including *GLRB*, which encodes the GlyR β-subunit (21, 22, 23, 24, 25). This subunit plays a major role in receptor trafficking and clustering at glycinergic synapses by interacting with the pleiotropic subsynaptic protein gephyrin (11, 26). In addition, the key amino acid residues R86 and E180 present in the GlyR β-subunit interact with ligand-binding residues present in the GlyR α-subunit via ionic interaction with the α-amino and carboxylate groups of bound glycine. As such, the function of low-affinity GlyR α_1_ subunit-containing mutants can be rescued by coexpression with the GlyR β-subunit, emphasizing a modulatory role of GlyR β in agonist binding (27, 28).

Several studies have reported recessive mutations in *GLRB* in different cases of hyperekplexia (23, 25, 28). The mutations cause improper folding of the GlyR β-subunit and altered trafficking of the whole protein, impacting the GlyR life-cycle, while reducing cell surface expression of the receptor (20, 29). As a result, the biophysical properties of currents mediated by activation of mutant GlyRs α_1_β heteromers are impaired (22, 28).

Here, we report the genetic and functional characterization of a novel variant in *GLRB* and its clinical outcome in a hyperekplexia patient. We found that A455P is located within a region important for maintaining the native 3D structure of the GlyR β-subunit. Expression of the mutation in a heterologous system resulted in reduced glycinergic currents and decreased levels of β-subunit protein at the plasma membrane. Overall, the findings help explain some of the phenotypic traits observed in our patient affected by hyperekplexia.

## Results

### Clinical features of patient II:3

This is a newborn girl, born to consanguineous parents, delivered normally at term by her 26-year-old mother. The father is 36 years old; parents are first cousins. An antenatal ultrasound scan was unremarkable except for excessive fetal movements. The mother reported that this pregnancy was marked by frequent abnormal movements compared to her previous two pregnancies. The Apgar score was 9 and 10 at one and five minutes, respectively. The birth weight of the newborn was 3.26 Kg (50th centile), and occipitofrontal circumference was 33 cm (below 10th centile). On routine neonatal examination, she was noticed to be stiff with sudden flexor spasms accompanied with apnea and oxygen desaturation. She was admitted to the neonatal intensive care unit for further evaluation and management. Clinical examination showed no dysmorphic features. She had an anxious looking face. Neurologic examination revealed increased tone, nonhabituating generalized flexor spasms to glabellar tapping, exaggerated startle reflex to loud sounds, and brisk deep tendon reflexes. Other system examinations were normal. A diagnosis of stiff-baby syndrome was made and she was started on clonazepam medication, to which she showed a good response. Her electroencephalogram and brain MRI were reported as normal. Other laboratory investigations including hematologic indices, liver functions, renal functions, urine organic acids, and lactate and ammonia levels were normal.

The patient was discharged from the neonatal intensive care unit at the age of 6 weeks. Upon follow-ups at the neurology clinic, she was still demonstrating an exaggerated nonhabituating response to glabellar tapping and loud sounds. She had a squint, and ophthalmic examination revealed left esotropia of 30 diopters by using the Hirschberg test. The Fundal examination was normal. She was prescribed glasses with patching of the right eye. She displayed hyperactivity, and her cognitive functions were moderately reduced. She is now attending normal school, but she is delayed in comparison to her peers. She still requires medication with clonazepam.

### Genetic analysis

**Figure 1:**
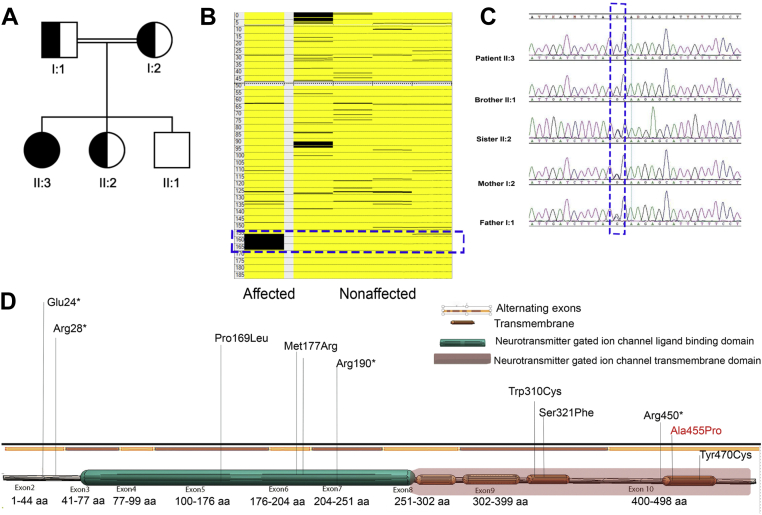
**A variant detected in a family with an autosomal recessive hyperekplexia case.***A*, the pedigree of the family shows the affected proband (*black*), the parents, and one sibling (sister) as carriers of the variant (*black* and *white*). *B*, the image shows the presence of the autozygous regions on chromosome 4. SNP data for the chromosome is arranged along the chromosome’s physical position. The homozygous calls are colored in *black*, and the heterozygous genotypes are in *yellow*. This allows the homozygous region common to the patient to be identified by the absence of *yellow* markers. The *left* column represents the patient’s genotypes, whereas the *right* columns consist of genotypes belonging to normal individuals in the family (father, mother, sister, and brother, respectively). The *black* region (pointed by the *dotted blue block*) in ch:4 (153–170 Mb) is a large homozygous region only present in the affected individual but not found in the other family members. There is a major hyperekplexia gene in this location *(GLRB*). *C*, the figure shows the chromatogram obtained from the sequencing of exon 10 on *GLRB*. The variation 1429G > C in the patient is shown where the guanine (G) is replaced with cytosine (C). *D*, annotated mutations in *GLRB* are displayed as a graphical view of the gene. The homozygous *GLRB* variant, A455P, is located within exon 10, identified in this study.

We studied a consanguineous Saudi family with an affected individual having hyperekplexia (Fig. 1). Her father, mother, and sister were found to be carriers based on genetic testing results (Fig. 1*A*). We performed a genome-wide SNP screening on GeneChip axiom arrays using blood from all the family members. The axiom arrays help to interrogate and then identify homozygous stretches in the human genome (SNP calls with AA or BB or both). Such stretches are frequently encountered through generations, particularly in consanguineous populations and utilized for genetic diagnosis of autosomal recessive diseases (30, 31). Our SNP analysis revealed a homozygous block (around 13 megabases in size) on chromosome 4 (Fig. 1*B*). The block (seen in black color solely in the patient’s column) consists of several genes including *GLRB* (Fig. 1*B*). We also performed next-generation sequencing (NGS) targeting exomes of a wide-range of neurological disorders and coupled it with autozygosity mapping to detect pathogenic variants in our patient. The results revealed a novel mutation within *GLRB* that is located in TM4 of the GlyR β-subunit (Fig. 1, *C* and *D*).

### Molecular modeling and predicted effect of A455P

**Figure 2:**
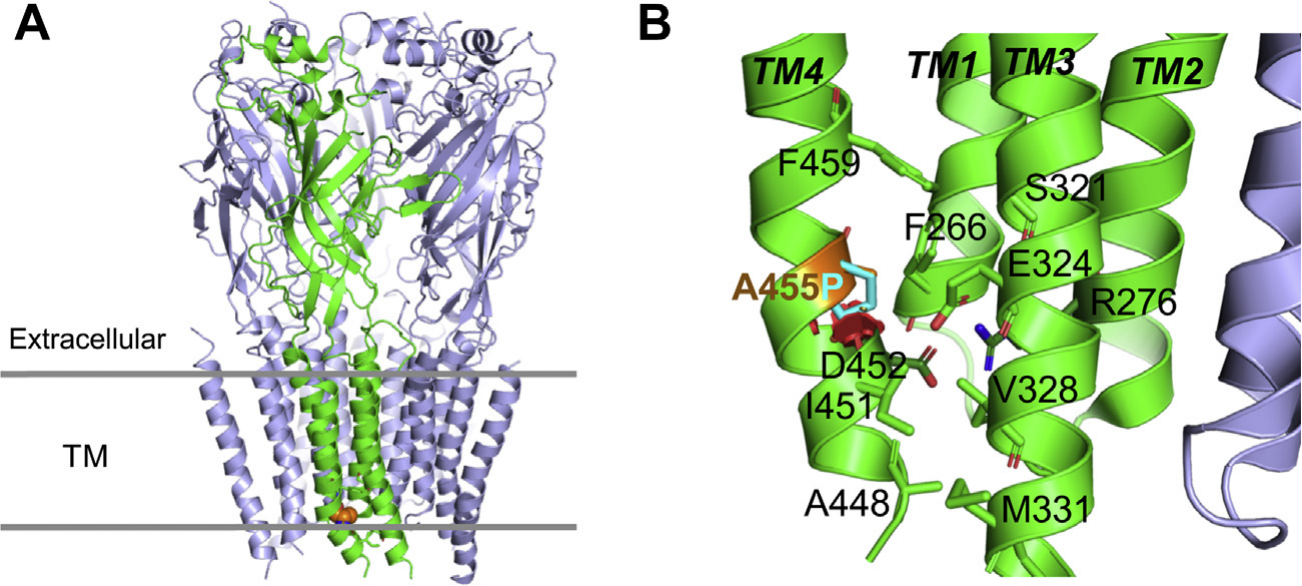
**Molecular environment and effect of the A455P missense mutation on the GlyR β-subunit.** The protein structure is based on the cryo-EM structure of native GlyR heteropentamer (PDB ID 7MLY). *A*, cartoon diagram of the pentamer side view. The β-subunit is colored in *green*. A455P is shown as *orange spheres*. The positions of the extracellular and transmembrame domains are indicated by *gray horizontal lines*. *B*, a zoomed-in view of the molecular environment of A455 (highlighted in *orange*). The substituting proline is shown as *cyan sticks*. Clashes caused by introduction of P455 are represented by *red discs* where the orientation and diameter show the direction of clashes, and the severity of clashes is illustrated by their thickness. Other key residues are shown in *stick* representation, and the TMs of the GlyR β-subunit are labeled. GlyR, glycine receptor; TM, transmembrane.

In the 4α_1_:1β heteropentameric GlyR, TM2 of the β-subunit lines one side of the ion channel. TM1, TM2, and TM3 contribute to the heterologous interactions with the four α subunits, while TM4 contacts TM1 and TM3 of the same β-subunit, sealing it against the membrane (12). A455 is located in the GlyR β TM4, toward the cytoplasmic side of the membrane, at a position where the N-terminal region of TM4 kinks markedly toward TM1 and TM3 (Fig. 2*A*). This kink allows the N-terminal region of TM1 (residues 448–455) to stay in contact with the other TMs and form hydrophobic interactions through A448, I451, and A455, and a buried ion bond between TM4 D452 and R276 from TM2. This ion bond is the only direct contact between TM4 and TM2 by providing a tether between TM4 and TM2. Hence the ionic bond is expected to have a direct effect on the channel. The introduction of a proline in position 455 leads to severe steric clashes with the backbones of I451 and D452 (Fig. 2*B*). In agreement, MutPred2 predicts that A455P weakens the helical conformation at this site (*p* = 0.29; *p*-value = 0.01). Consequently, the A455P mutation is expected to destabilize the TM region close to the cytoplasm and affect the position and dynamics of TM2. Thus, the A455P variant is predicted to affect the channel permeability. In support, A455P was predicted to be possibly damaging by affecting the protein’s function by PolyPhen-2 (score = 0.582; sensitivity = 0.88; specificity = 0.91), Sorting Intolerant From Tolerant (score = 0.02), and PANTHER (preservation time = 361, possibly damaging, score 0.5). The Combined Annotation Dependent Depletion (CADD), MutationAssessor, and MutationTaster also predicted this missense change to be disease causing.

By affecting the stability and channel conductance of GlyR, A455P may produce similar effects to those observed for truncations in the intracellular TM3-4 loop in individuals affected by hyperekplexia (21) or in the mouse mutant *oscillator* (50), causing a loss of function of GlyRs.

### Differences in GlyR staining in cells expressing the A455P variant

**Figure 3:**
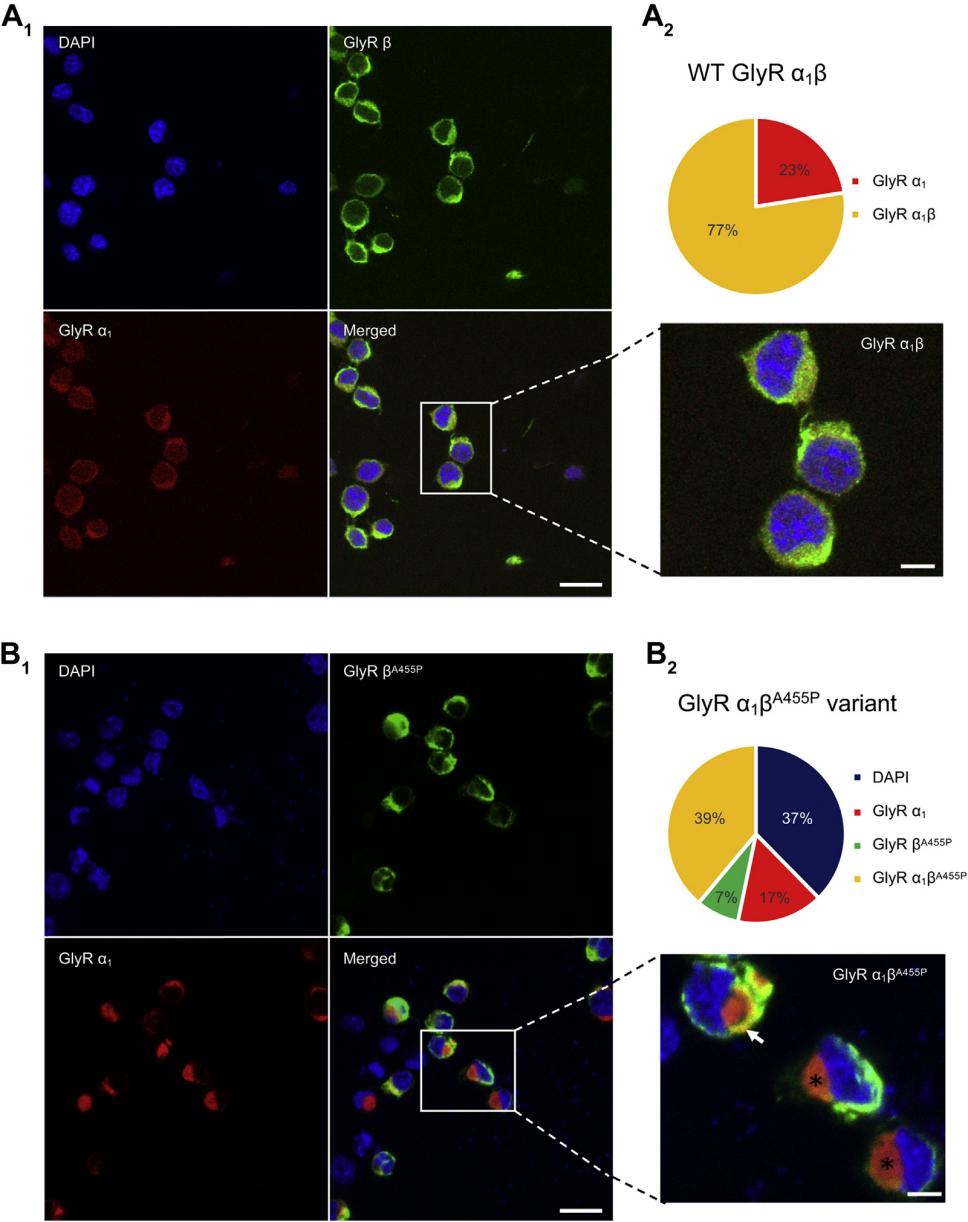
**Confocal imaging of WT and mutant GlyR subunits in N2A cells.** Triple immunofluorescence staining of nucleus (DAPI, *blue*), GlyR β-subunit (Alexa Fluor 488, *green*), and GlyR α_1_ (mRFP, *red*) in N2A cells visualized with laser scanning confocal microscopy. *A*_*1*_, WT, exemplar confocal microscopy images (three channels plus merged) showing widespread expression of GlyR α_1_ and β-subunits in cytosolic and plasma membrane areas of the cells (inset). *A*_*2*_, proportion of cells stained for GlyR subunits as a fraction of the total number of DAPI-positive cells. *B*_*1*_, mutant, immunostaining of GlyR α_1_ and β^A455P^ subunits. Some cells are void of GlyR α_1_- or β-subunit staining (merged). The GlyR α_1_-subunit is mainly confined to cytosolic areas and remains segregated from the β-subunit (inset, *asterisks*). *Yellow patches* can be found in putative plasma membrane regions (inset, *arrow*). *B*_*2*_, proportion of cells stained for GlyR α_1_, GlyR β^A455P^, and GlyR α_1_β^A455P^. Calibration bars in A_1_, B_1_ (20 μm, insets: 5 μm). All images are from single optical z-sections using a 40x objective. GlyR, glycine receptor.

To investigate the effect of A455P on cellular distribution of GlyRs, we performed immunohistochemical labeling of GlyR subunits and imaged their localization in N2A cells. Confocal imaging of WT GlyR subunits demonstrated GlyR α_1_ and GlyR β in cytosolic and putative plasma membrane regions (Fig. 3*A*_1_). Fluorescence signals from GlyR α_1_ and GlyR β-subunits overlapped in a majority of cells, suggesting a widespread expression of both subunits (Fig. 3*A*_1_, inset). Cells transfected with and stained for GlyR α_1_ and mutant GlyR β^A455P^ subunits, however, clearly demonstrated a GlyR expression profile distinct from WT and a segregated pattern of subunit localization. The GlyR α_1_-subunit was mainly confined in the cytoplasm, whereas GlyR β^A455P^ could be found both in cytosolic and plasmalemmal areas of the cell (Fig. 3*B*_1_). However, some cells demonstrated yellow patches in plasmalemmal regions as found in cells expressing the WT receptor (Fig. 3*B*_1_, inset).

In order to quantify differences in staining profile between the WT and mutant, a count of the number of cells expressing the different GlyR subunits was performed. In the WT, 77% of DAPI-positive cells expressed GlyRs α_1_ and β-subunit, and 23% expressed GlyR α_1_-subunit only (Fig. 3*A*_2_). In contrast, a similar count performed in cells expressing mutant GlyRs showed that 39% were stained for GlyR α_1_ and β^A455P^ and that 17% showed GlyR α_1_ and 7% GlyR β^A455P^ only (Fig. 3*B*_2_). Notably, in 37% of cells transfected with mutant GlyRs, we were not able to detect protein expression of GlyR α_1_ or GlyR β^A455P^ subunits. Thus, our immunohistochemical labeling of GlyR subunits showed noticeable differences in staining in the A455P group compared to that of the WT.

### A455P reduces glycine sensitivity and maximum currents

**Figure 4:**
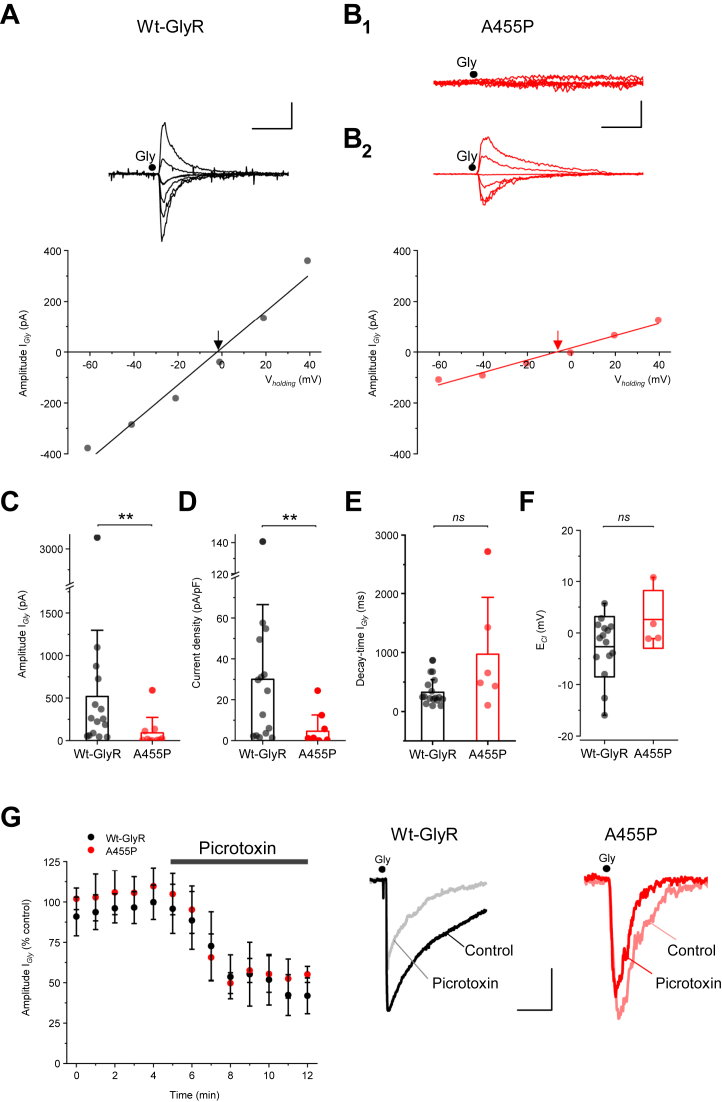
**Electrophysiology and pharmacology of WT and mutant GlyRs.***A*, *top*, typical example of glycinergic currents in a cell expressing WT GlyRs α_1_β. Glycine puffs are as indicated (5 ms, 7 psi). *Bottom*, I-V relation in the same cell, V_*holding*_ = -60 to +40 mV, increment 20 mV. Fit of I-V relation indicated by *solid-line*, arrow indicates E_Cl_ (-2.7 mV). *B*_*1*_ and *B*_*2*_, collection of traces taken from two different cells expressing mutant GlyRs α_1_β^A455P^. *B*_*1*_, no current is detected in one cell in response to glycine application at various membrane potentials (V_*holding*_ = -60 to +40 mV, increment 20 mV). *B*_*2*_, glycinergic currents in a different cell expressing the A455P mutation where an I-V protocol is performed (E_Cl_ = -6.7 mV). Calibration bars: 200 pA, 200 ms. *C*–*F*, summary bar plots (mean ± SD) depicting the amplitude, current density, and decay time of glycine-evoked currents, and E_Cl_ measurements (box plot is mean ± SD, *whiskers* represent min and max). Individual data points are shown in the overlay. A Kruskall–Wallis ANOVA showed a significant difference in current amplitude (Χ^2^ (2) = 8.97, *p* = 0.003) between WT and mutant GlyRs, with a mean rank of amplitude score of 16.6 for GlyRs α_1_β (n = 15) and 7.6 for α_1_β^A455P^ (n = 10). Similar results were found for the current density (Χ^2^ (2) = 9.64, *p* = 0.002), with a mean rank of 16.7 for GlyRs α_1_β (n = 15) and 7.4 for α_1_β^A455P^ (n = 9). The decay time of glycinergic currents is unaffected (Χ^2^ (2) = 2.9, *p* = 0.086) with a mean rank of 9.53 for GlyR (n = 15) and 14.6 for GlyR α_1_β^A455P^ (n = 6). Summary box plots of E_Cl_ show no difference. Χ^2^ (2) = 1.69, mean rank for GlyR α_1_ = 9.13 (n = 15) and for GlyR α_1_β^A455*p*^ = 13.3 (n = 4; *p* = 0.086). *G*, *left*, plots of glycine-evoked current amplitude against time showing a partial reduction in the presence of picrotoxin. No difference in picrotoxin sensitivity is observed between groups (WT, n = 6; A455P, n = 3; *p* = 0.89, unpaired *t* test). *Right*, averaged current traces (10 consecutive trials) taken before and after the application of picrotoxin (50 μM) onto cells expressing WT (*black*) or mutant GlyRs (*red*). Calibration bars: WT, 200 pA, 100 ms; A455P: 20 pA, 200 ms. The Y-axis in C and D is truncated for clarity. All data are presented as mean ± SD. ∗∗*p* < 0.01, Bonferroni correction for multiple tests. GlyR, glycine receptor.

Previous reports have demonstrated the impact of *GLRB* mutations on the functionality of GlyRs (22, 24, 28). A reduction in peak currents was found for the homozygous missense substitution L285R, with no change in glycine affinity (28), whereas the G229D and M177R missense substitutions yielded low-affinity GlyRs compared to WT (22, 28). To analyze the effect of A455P on activation of GlyRs, we obtained recordings from N2A cells acutely transfected with the WT or mutant GlyRs. All cells transfected with GlyRs α_1_ and β responded to glycine application with robust currents (Fig. 4*A*). In stark contrast, 40% of cells expressing mutant β^A455P^ showed no current in response to glycine puff (Fig. 4*B*_1_). The remaining cells exhibited glycinergic currents, the amplitude of which varied between 24.1 and 591.7 pA (mean ± SD, A455P: 216.1 ± 127.5 pA, n = 6; Fig. 4*B*_2_). Over the entire cell population, the mean amplitude of glycinergic currents was reduced in the mutant group (WT: 519.1 ± 778.9 pA, n = 15; A455P: 90.5 ± 182.7 mV pA, n = 10, *p* = 0.002; Fig. 4*C*). Similar results were obtained for current density (WT: 29.9 ± 36.6 pA/pF, n = 15; A455P: 4.5 ± 8 pA/pF, n = 10; *p* = 0.001; Fig. 4*D*). However, the decay time constant of glycinergic current was not significantly affected by the mutation (WT: 325.4 ± 215.3 ms, n = 15; A455P: 969.9 ± 963.8 ms, n = 6; *p* = 0.08; Fig. 4*E*). Finally, we calculated E_Cl_ in cells that responded to glycine and found no discernible difference between the mutant and WT receptors (WT: -2.2 ± 1.7 mV, n = 15; A455P: 2.6 ± 2.8 mV, n = 4; *p* = 0.19; Fig. 4*F*). Thus, the A455P mutation reduces glycinergic currents but has no effect on the Cl^-^ selectivity of the receptor ionophore.

One explanation for the presence of glycinergic currents in cells expressing mutant GlyRs is that, they might be expressing homomeric GlyRs α_1_ mainly and little or no GlyR α_1_β heteromers. To address this possibility, we tested the effect of picrotoxin, whose IC_50_ for homomeric GlyRs α_1_ (5–10 μM) is 50 to 100 fold less than that of heteromeric GlyRs αβ (32). Picrotoxin (50 μM) added to the perfusion solution onto cells expressing WT GlyRs α_1_β reduced the amplitude of glycine-evoked currents by 41 ± 15.6% (n = 6). This effect was no different to the decrease in current amplitude observed in cells expressing mutant GlyRs α_1_β^A455P^ (44.6 ± 9.9% reduction, n = 3; Fig. 4*G*).

**Figure 5:**
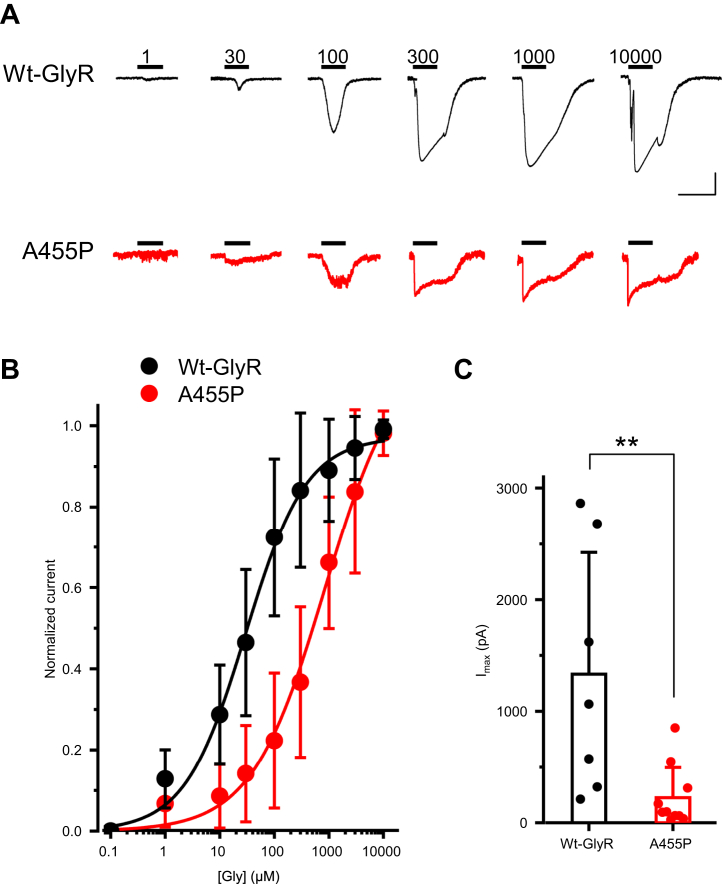
**Concentration–response relationship.***A*, example current traces obtained in acutely transfected N2A cells expressing WT GlyR α_1_β (*black*) or GlyR α_1_β^A455P^ (*red*) and exposed to increasing concentrations of glycine (*horizontal bars*), as indicated (μM). Calibration bars: WT, 1000 pA, 500 ms; A455P: 100 pA, 500 ms. *B*, normalized concentration–response curves for WT GlyRs α_1_β (*black*) and mutant α_1_β^A455P^ (*red*) fitted with the Hill equation (shown as a *continuous line*). Fitted parameters for WT GlyR α_1_β are as follows: EC_50_, 29.7 ± 3.8 μM; nH, 0.77 ± 0.08 (n = 7); and those for mutant GlyR α_1_β^A455P^ are as follows: EC_50_, 843.9 ± 305.3 μM; nH, 0.64 ± 0.07 (n = 10). *C*, bar plot of peak-current amplitude (I_max_) at a saturating concentration of glycine (10 mM). GlyR α_1_β (n = 7) and GlyR α_1_β^A455P^ (n = 10). All data are represented as mean ± SD. ∗∗*p* < 0.01, Mann–Whitney U test. GlyR, glycine receptor.

Next, we investigated whether the A455P mutation affects agonist binding affinity and efficacy. We obtained concentration–response curves from peak currents elicited by the application of a range of glycine concentrations (Fig. 5*A*). EC_50_ values for GlyR α_1_β and GlyR α_1_β^A455P^ were 29.7 ± 3.8 and 843.9 ± 305.3 μM, respectively, with little effect upon the Hill coefficient (Fig. 5*B*). Furthermore, currents elicited by a saturation concentration of Gly (10 mM) were smaller for the A455P mutant (I_*max*_, 225.8 ± 271.6 pA, n = 10) than those of the WT (1332.3 ± 1092.3 pA, n = 7, *p* = 0.005; Fig. 5*C*). These results demonstrate that A455P reduces glycine sensitivity and maximum current.

### A455P decreases GlyR β-subunit levels and preserves coassembly with GlyR α_1_

**Figure 6:**
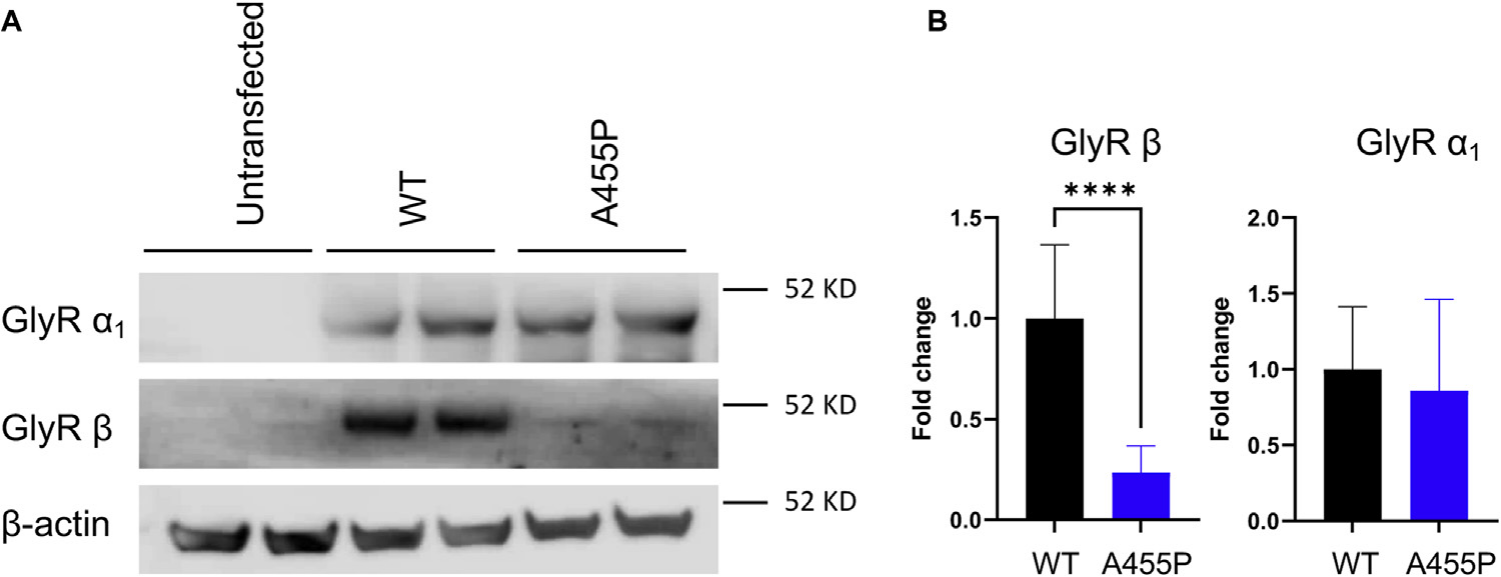
**Protein expression of the mutant A455P GlyR β-subunit is reduced in a heterologous model system.** Untransfected N2A cells or N2A cells coexpressing the GlyR α_1_-subunit with the WT or mutant A455P GlyR β-subunit were lysed and probed for Western blotting. *A*, immunoblot image of GlyR β and GlyR α_1_-subunit expression in N2A cells. *B*, densitometric analysis of WT and mutant GlyR β binding to GlyR α_1_ (n = 4). Subunit expression of GlyR β and GlyR α_1_ in cells cotransfected with the WT proteins is compared with the expression levels in cells cotransfected with the A455P GlyR β-subunit. For quantification, protein expression was adjusted to the loading control β-actin. The GlyR β-subunit was normalized to the corresponding input. The data are represented as mean ± SD. ∗∗∗∗*p* < 0.0001, unpaired *t* test. GlyR, glycine receptor.

To gain mechanistic insights into the effect of the A455P mutation on subunit interactions, trafficking, and membrane localization, we performed immunoblot assays on the whole-cell lysate and isolated membrane fraction following acute transfection of GlyRs in N2A cells. We first determined the impact of the A455P mutation on GlyR β expression levels using Western blots. As shown in Figure 6, the amount of GlyR β protein expression was significantly reduced to approximately a quarter in whole cells cotransfected with GlyR α_1_ and mutant GlyR β^A455P^ in comparison to cells expressing the WT receptor. The isolated membrane fraction also showed reduced expression of mutant GlyR β^A455P^ protein in comparison to WT GlyR β (Fig. S1, input).

Next, we examined whether the GlyR α_1_-subunit interacts with the mutant GlyR β^A455P^ to form heteromeric receptors by performing coimmunoprecipitation experiments on cell lysate using anti-GlyR α_1_ antibodies. Results indicated the assembly of heteromeric GlyRs in the cytoplasm for WT and mutant receptors with a nonsignificant trend indicating a possible increased affinity of the GlyR β^A455P^ subunit toward GlyR α_1_ in comparison to the WT receptor (Fig. S2). Coimmunoprecipitation experiments using the membrane fraction showed very little difference between untransfected cells and experimental GlyR subunit transfections, preventing further interpretation on subunit assembly in the membrane using this assay (Fig. S1, IB).

## Discussion

The present study focuses on the identification of hyperekplexia in individuals of Saudi origin. By collaborating with neurologists, paediatricians, and clinical geneticists, we have been able to identify a disease-causing variant in a patient from a consanguineous family. Our approach revealed the novel variation c.1429G > C (A455P), within *GLRB*, which encodes the GlyR β-subunit. Firstly, we found that A455 is located within a region of the GlyR, critical for protein stability and ion channel structure. A proline in this position would destabilize the TM region and may alter the shape and hence conductance of the channel. Secondly, A455P lowered the amount of GlyR β protein found in the cytoplasm and membrane. Thirdly, we demonstrated that the mutant GlyR β^A455P^ coassembled with GlyR α_1_ and that expression of both subunits in a heterologous system yielded GlyRs with reduced agonist sensitivity and efficacy but had no effect on the ion selectivity of the associated channel.

Our molecular modeling predicted that A455P destabilizes the native helix conformation of TM4 and its attachment to the rest of the trans-membrane protein core. Introduction of a proline at this position would lead to steric clashes, destabilizing TM4 and its attachment to the protein core. The effect of A455P was predicted to be analogous to that of variants of the intracellular loop linking TM3 and TM4, or in the TM4 region of GlyRs, as previously reported in hyperekplexia cases. Such variants resulted in misorientation of the entire TM4 and defective trafficking of GlyRs to the cell surface (20, 24).

Our immunohistochemical labeling of GlyR α_1_, β, and β^A455P^ subunits showed a distinct staining profile in the mutant group compared to WT. This result is in line with previous reports highlighting the detrimental effect of *GLRB* mutations on cellular trafficking and localization of GlyRs (24, 28). Although GlyR β^A455P^ was detected in cytosolic and putative plasmalemmal regions of the cell, the GlyR α_1_-subunit remained confined to cytosolic regions where it might undergo degradation, which in turn could affect its trafficking and insertion into the plasma membrane. Alternatively, expression of the mutant β-subunit could reduce overall levels of GlyR subunits within the cell, yielding fewer heteromers. Using quantitative Western blot analysis, we confirmed that the A455P mutation decreases the amount of GlyR β-subunit within the cell. Coassembly with the GlyR α_1_-subunit is maintained and intersubunit affinity is possibly increased. However, we cannot exclude the possibility that the mutant GlyR β-subunit also interacts with unrelated membrane proteins. Overall, the data indicate that less heteromeric GlyRs reach the cell membrane and that these receptors might have different intermolecular subunit interactions, resulting in the observed electrophysiological changes. Thus, at native glycinergic synapses, A455P is likely to affect the number of heteromeric GlyRs, the assembly of GlyR α_1_ and β-subunits, and the anchoring of heteromeric GlyRs to postsynaptic sites, impacting on the strength of synaptic inhibition.

We found that a proportion of cells expressing mutant GlyRs exhibited glycinergic currents. Again, this finding indicated that β^A455P^ subunits could coassemble with GlyR α_1_ to form membrane-bound receptors, a finding that was substantiated by our biochemical analysis showing α_1_β^A455P^ coassembly. However, at a saturating concentration of glycine, maximum currents recorded from cells expressing the A455P mutation were smaller in comparison to WT. Current density was also reduced, in line with previous electrophysiological analysis of homozygous missense mutations L285R and M177R identified in *GLRB*, showing a reduction in glycine-evoked peak currents (28). Other missense mutations reported as R450X and Y470C, assayed with EYFP fluorescence quenching, also reduced chloride fluxes, indicating decreased GlyR activity (24, 28). A glycine concentration–response relationship obtained for the L285R mutant indicated that agonist sensitivity for GlyRs was unchanged. However, L285R was associated with spontaneously opening GlyR channels (28). A number of hyperekplexia-like mutations introduced in the TM2-TM3 loop of the GlyR β-subunit were also shown to be uncoupled from changes in channel gating (33). Our concentration-response curves, however, showed reduced agonist sensitivity in cells expressing GlyR α_1_β^A455P^, which is in line with the effect of previously reported substitutions M177R and G229D in the β-subunit, also known for decreasing glycine sensitivity and efficacy (24, 28). The observed reduction in apparent glycine efficacy for the mutated receptor is consistent with the view that A455P located in TM4 disrupts intermolecular interactions that are important for receptor activation and gating (27). During the review of this manuscript, a second study (34) reported functional analysis of three GlyR β-subunit mutations: Y252S, S321F, and A455P. In contrast to this study, the authors reported that α_1_β^A455P^ GlyRs displayed increased maximal currents that were not accompanied by enhanced surface expression or changes in EC_50_ values. Normalization of the dose-response curve to the maximal currents obtained from α_1_β suggested a gain-of-function for α_1_β^A455P^. However, transfection of GlyR β^A455P^ into hippocampal neurons revealed a significant reduction in GlyR β-positive synapses, consistent with a loss of synaptic signalling.

Finally, we tested the effect of picrotoxin and found no difference between cells expressing WT or mutant GlyRs. This result was in good agreement with the similar sensitivity to picrotoxin between WT GlyR α_1_β and mutant GlyR α_1_β^G229D^ (22). Overall, our picrotoxin data suggested that the missense mutation A455P did not alter the ratio of heteromeric versus homomeric GlyRs, consistent with a global decrease in localization of both GlyR α_1_ and β-subunits at the cell membrane.

In summary, our genetic screening yielded a novel variant within *GLRB*. High resolution cellular imaging and functional characterization of the variant validated a loss-of-function mutation whose pathogenicity is likely to cause hyperekplexia in the affected individual.

## Experimental procedures

### Ethical statement

Clinical diagnosis of hyperekplexia was ascertained by referral from neurologists, pediatricians, or clinical geneticists from the King Faisal Specialist Hospital and Research Center. The patient and remaining family members (Fig. 1*A*) were consented under King Faisal Specialist Hospital and Research Center IRB-approval protocol (RAC # 2120022 and 2180004). Studies in this work abide by the Declaration of the Helsinki principles.

### DNA isolation, PCR, and Sanger sequencing

DNA was isolated from intravenous blood samples using the PureGene DNA Purification Kit (Gentra Systems, Inc). The quality and quantity were determined using NanoDrop ND-1000 (Nano Drop Technologies Inc). Primers designed by using a Primer3 web-based tool were optimized on human control DNA. PCR was performed according to standard protocols. PCR products were sequenced using the Sanger DNA sequencing protocol using the 3730 XL Analyzer (Applied Biosystems). The sequencing results were analyzed using the ChromasPro (Technelysium Pty Ltd) and the SeqMan software (https://www.dnastar.com/software/lasergene/seqman-ngen).

### Genome-wide SNP genotyping and autozygome analysis

SNP genotyping was performed using GeneChip Human Genome-wide SNP Axiom Arrays according to the manufacturer’s protocols and guidelines (Affymetrix Inc). Generated SNP calls were used for autozygosity mapping using AutoSNPa software (http://dna-leeds.co.uk/autosnpa/) as previously described (35, 36).

### Gene panel screening using NGS

A comprehensive NGS gene panel (Neuropanel) was developed and used as previously published (PMID: 26112015). Briefly, the Neuropanel covers the exons and flanking regions of 756 genes that are implicated in genetic disorders involving in neurological diseases. The panel screening was performed on DNA samples using the Ion Proton System (Life Technologies). A PCR library of the coding and surrounding sequences of the genes in the Neuropanel was established according to the published protocol (PMID: 26112015). Primers were designed based on an average size of amplicons (∼210 bp exons and flanking sequences) with at least 90% coverage of the targeted exons. DNA samples were amplified using the designed primers in pools. Amplified DNA fragments were pooled, digested with FuPa reagent (Thermo Fisher Scientific), and then ligated to predesigned adapters. The samples were purified and normalized to 100 pM. The normalized libraries were barcoded and used for emulsion PCR on an Ion OneTouch System and finally sequenced on an Ion Proton instrument (Thermo Fisher Scientific).

### Immunocytochemistry and confocal microscopy

For immunocytochemistry experiments, each plate was transfected with either 2.8 μg of pCMV6-AC-mRFP-GlyR α_1_+pRK5-GlyR β or pCMV6-AC-mRFP-GlyR α_1_+pRK5-GlyR β^A455P^ and left for 48 h at 37 °C. All transfections made use of the FuGENE HD transfection reagent (Promega) and were carried out in N2A cells grown on 6 cm petri dishes. The transfection mixture was incubated at room temperature for 15 min and subsequently added to the cells. Following permeabilization, cells were incubated overnight at 4 °C with the primary antibody (GlyR mouse monoclonal antibody; Santa Cruz Biotechnology) at 1:200 and 1:300 dilutions. Cells were then incubated for 1 h at room temperature with a 1:500 fluorescent secondary antibody (Alexa Fluor 488, goat anti-mouse IgG from Life technologies). A 1:1000 dilution DAPI antibody (Thermo Fisher Scientific) was incubated in blocking buffer for 30 min at room temperature to label the nucleus. We also performed immunocytochemistry control experiments in which we omitted the primary antibody specific for the GlyR β-subunit or where constructs with no fluorescent fused protein were expressed (Fig. S3).

For laser scanning confocal microscopy, images were acquired with a ZEISS LSM 710 confocal microscope using a x40 (1.4 NA) objective and immersion oil. Images were obtained from three independent transfections and staining using the ZEN software (https://www.zeiss.com/microscopy/int/products/microscope-software/zen.html). Cell counting was performed using the Image J Fiji plugin on maximum fluorescence intensity projections of Z-stacks. The number of DAPI-positive nuclei was used as the total number of cells per image. The number of mRFP and Alexa Fluor 488–positive cells was calculated as a ratio of the total number of DAPI-positive cells.

### Plasmid preparation, cloning, and site-directed mutagenesis

Variants were introduced into the pRK5-GlyR β using the QuikChange site-directed mutagenesis kit (Agilent Technologies). All expression constructs were confirmed by Sanger sequencing of the entire coding region. GlyR α_1_ and GlyR β-subunit expression constructs were transfected at a DNA ratio of 1:5 to promote the formation of heteromeric GlyRs α_1_β. After 48 to 72 h, cells were used for immunocytochemistry or electrophysiology.

### Electrophysiology

N2A cells acutely transfected with GlyRs α_1_β (WT) or α_1_β^A455P^ (variant) and eGFP were placed in a recording glass chamber continuously superfused with saline solution containing the following (in mM): NaCl (140), KCl (4), D-glucose (5), CaCl_2_ (2), MgCl_2_ (6), Hepes (10) (pH 7.2, osmolarity 298 mOsmol.l^-1^). Patch pipettes were pulled from borosilicate glass capillaries with filament (outer diameter 1.5 mm, 0.5 mm wall thickness, Warner Instruments), which had a resistance of 3 to 4 MΩ. Recordings were obtained from eGFP-positive N2A cells under infrared differential interference contrast imaging at 22 °C. The pipette solution contained the following (in mM): KCl (155), NaCl (8), EGTA (10), MgCl_2_ (4), MgATP (0.3), Na_3_GTP (0.3), Hepes (10), Na_2_-phosphocreatine (10). Glycine (10 mM, in saline solution) was pressure-applied (5–20 psi, 5–10 ms) to eGFP-positive cells *via* a patch pipette connected to a Picospritzer (General Valve Corporation). The pipette was approached at a distance ranging from 50 to 200 μm from the cell membrane and placed such that pressure ejection of glycine followed the stream of the perfusion solution. With this arrangement, the cell was always exposed to glycine as a result of the application. For the characterization of dose–response curves, a pipette with a larger open-tip diameter (100–200 μm) was connected via supply lines to reservoirs (37) filled with perfusion solution containing different concentrations of glycine (0.1–10,000 μM). Solution exchange was operated through the action of computer-controlled pinch-valves (NanIon). Whole-cell currents were recorded with a Multiclamp 700 B amplifier (Molecular Devices), filtered at 2 kHz (internal four-pole low-pass Bessel filter), and sampled at 10 kHz. The series resistance was <15 MΩ, and results were discarded if it varied >20%. Glycine puffs were delivered every 20 s at different holding potentials (−60 to +40 mV, 20 mV increment) to characterize the current-voltage (I-V) relation in glycine-responsive cells, after which picrotoxin (Sigma-Aldrich, 50 μM in DMSO) was applied at V_*holding*_ = −60 mV.

### Western blotting

GlyR α_1_ and GlyR β-subunit expression constructs were transfected at a DNA ratio of 1:1 into N2A cells using Fugene (Promega). After 48 h, cells were collected using cell lysis buffer (150 mM NaCl, 50 mM Tris pH 7.5, 5 mM EDTA and 0.25% nonyl-phenoxypolyethoxylethanol-40, NP-40) containing protease inhibitor (Roche) and Halt phosphatase inhibitor (Thermo Fisher Scientific). Membrane proteins were collected using a Membrane Protein Extraction Kit (Thermo Fisher Scientific) according to the manufacturer’s instructions. Equal amounts of protein were loaded on premade gels, Bolt 4 to 12%, Bis-Tris (Thermo Fisher Scientific) followed by transfer of proteins onto polyvinylidine fluoride membranes (Biorad). Polyvinylidine fluoride membranes were blocked for 1 h in Tris-buffered saline, 0.1% Tween 20 with 5% (w/v) nonfat dry milk. Primary antibodies against GlyR α_1_ (1:1000, 17951-1-AP, Proteintech), GlyR β (1:500, SC-365819, Santa Cruz), Anti-β-Actin (1:2000, A2228, Sigma), and Na, K-ATPase (1:1000, #3010, Cell Signaling Technology) were incubated overnight at 4 °C. After three 10 min washes with Tris-buffered saline, 0.1% Tween 20, secondary antibodies against rabbit (1:5000, #111-035-003, Jackson ImmunoResearch) or mouse (1:5000, #115-035-003, Jackson ImmunoResearch) were added and incubated further for 1 h at room temperature. After another three 10 min washes, the chemiluminescent assay was developed using SuperSignal West Pico/Femto Chemiluminescent HRP Substrate (Thermo Fisher Scientific). The SynGene GeneGnome imaging system was used for image acquisition and quantification.

### Coimmunoprecipitation

Cells were collected in the same way as for Western blot experiments. A Dynabeads Protein G for immunoprecipitation kit was used in accordance with the modified manufacturer’s instructions as follows: 50 μl Dynabeads were suspended in 200 μl PBS with 0.02% tween containing 5 μg of GlyR α_1_ antibody and rotated for 10 min. The Dynabeads-antibody complex was washed three times with PBS, and 100 μl of cell lysate was added. The mixture was rotated for 2 h and exposed to a magnet. Extensive washing with PBS was performed, followed by the addition of 20 μl of 50 mM glycine pH 2.8 (elution buffer) and 10 μl of NuPAGE LDS sample buffer with NuPAGE reducing agent mix. At this point, a Western blot protocol was carried out as outlined above. The primary antibodies were the same as those used in Western blot experiments.

### Data analysis and statistical tests

Data acquisition and analysis were performed using customized virtual instruments programmed in LabVIEW (V8.0, https://www.ni.com/en-in/shop/labview.html). Results from the analysis were exported into Origin Pro (2019, https://www.originlab.com/2019) for figures’ production. To calculate the reversal potential for GlyRs (E_Cl_), the intercept between the linear fit of the I-V relation and the x-axis was used. Junction potentials were not corrected. Current density, which provides an estimate of the number of functional GlyRs per μm^2^ of cell surface membrane area, was calculated by dividing the amplitude of the mean glycinergic current (V_*holding*_ = −60 mV) by whole-cell capacitance. The decay times of glycine currents were determined by fitting a single exponential to the (90–10%) decay phase. Concentration–response curves were fit using the Hill equation, I/Imax = A_1_ + (A_2_ – A_1_)/(1 + 10(logEC_50_ − log[agonist]) × Hill slope), where A1 and A2 refer to top and bottom asymptotes, I is the current amplitude activated by a given concentration of glycine, Imax is the maximum response of the cell, and EC_50_ is the concentration eliciting a half-maximal response. In pharmacological experiments, the amplitude of glycinergic currents was averaged over three successive trials and normalized to the predrug amplitude level to obtain a timecourse. Hypothesis testing and statistical inferences from the data were performed using the IBM SPSS Statistics 26 software (https://www.ibm.com/support/pages/downloading-ibm-spss-statistics-26). Data were subjected to normality distribution tests before performing a statistical analysis using parametric or nonparametric tests. Data are expressed as mean ± SD and were considered significant if *p* < 0.05.

### Computational structural analysis of the A455P variant

The 3D structure of the GlyR β-subunit in the context of the native 4α_1_:1β pentameric assembly was retrieved from the protein data bank (PDB) (38) based on the cryo-EM structure of native GlyR oligomers (PDB ID 7MLY) (12). RaptorX (39) was used for predicting secondary structure and disorder. The mutation was evaluated using the Pymol program (http://www.pymol.org).

### *In silico* prediction analysis

The variant pathogenicity was predicted using MutPred algorithms (40), Polyphen-2 (41), Sorting Intolerant From Tolerant (42), PANTHER (43), MutationAssessor (44), CADD (45), and MutationTaster (46, 47).

## Web sources

http://browser.1000genomes.org/index.html

http://evs.gs.washington.edu/EVS/

http://www.ncbi.nlm.nih.gov/projects/SNP/

https://www.ncbi.nlm.nih.gov/clinvar/

http://www.hgmd.cf.ac.uk/docs/login.html

http://gnomad.broadinstitute.org/

http://exac.broadinstitute.org/

## Data availability

Data supporting our findings can be found in the text, figures, and supporting information.

## Supporting information

This article contains supporting information.

## Conflict of interest

The authors declare that they have no conflicts of interest with the contents of this article.
