# Supplementary material for: Clinical, genetic, and functional characterization of the glycine receptor β-subunit A455P variant in a family affected by hyperekplexia syndrome
Source: J Biol Chem. 2022 May 6;298(7):102018. doi: 10.1016/j.jbc.2022.102018 (PMC9241032; doi:10.1016/j.jbc.2022.102018)
Supplement: Supplemental Figure S1 [file mmc1.pdf]

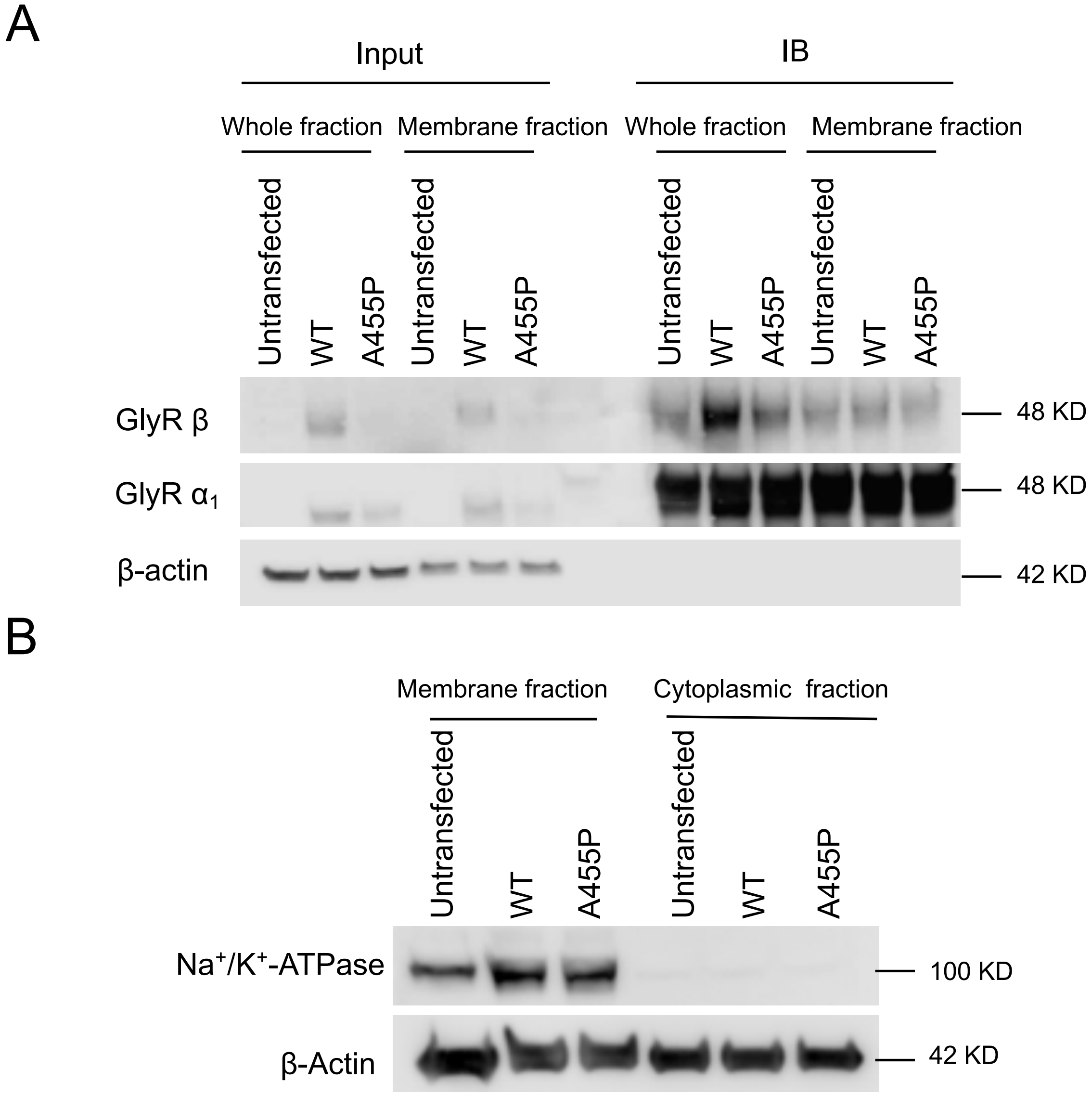

Fig. S1. Expression of mutant GlyR β^{A455P} and co-assembly with GlyR α$_1$ at the plasma membrane. *A*, immunoblot of the GlyR β-subunit immunoprecipitated using GlyR α$_1$ antibodies in whole cell and plasma membrane fractions of N2A cells co-expressing GlyR α$_1$ with wild-type or mutant GlyR β-subunit. Inputs are immunoblots of the same protein in cell lysates and membrane fraction before co-immunoprecipitation. *B,* immunoblot showing successful plasma membrane fractionation.
