# Supplementary material for: Clinical, genetic, and functional characterization of the glycine receptor β-subunit A455P variant in a family affected by hyperekplexia syndrome
Source: J Biol Chem. 2022 May 6;298(7):102018. doi: 10.1016/j.jbc.2022.102018 (PMC9241032; doi:10.1016/j.jbc.2022.102018)
Supplement: Supplemental Figure S2 [file mmc2.pdf]

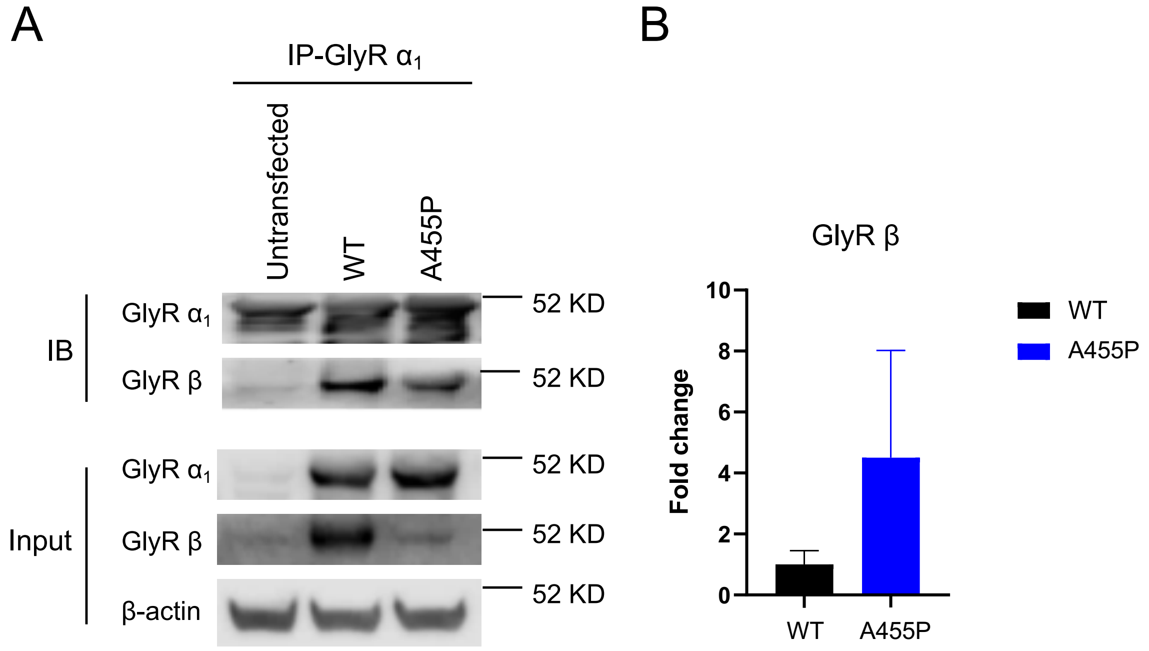

Fig. S2. The mutant A455P GlyR β-subunit co-assembles with the GlyR α_1-subunit in a heterologous expression system. *A,* GlyR β was purified using an anti-GlyR α_1 antibody in N2A cells co-expressing GlyR α_1 with wild-type GlyR β or mutant GlyR β^{A455P} and the co-precipitating proteins were detected by immunoblotting. Inputs are immunoblots of the same protein in cell lysates before co-immunoprecipitation. *B,* quantification of wild-type and mutant GlyR β binding to GlyR α_1 (n = 4). The GlyR β-subunit was normalized to the corresponding input. Data are represented as mean ± SD.
