# Supplementary material for: Clinical, genetic, and functional characterization of the glycine receptor β-subunit A455P variant in a family affected by hyperekplexia syndrome
Source: J Biol Chem. 2022 May 6;298(7):102018. doi: 10.1016/j.jbc.2022.102018 (PMC9241032; doi:10.1016/j.jbc.2022.102018)
Supplement: Supplemental Figure S3 [file mmc3.pdf]

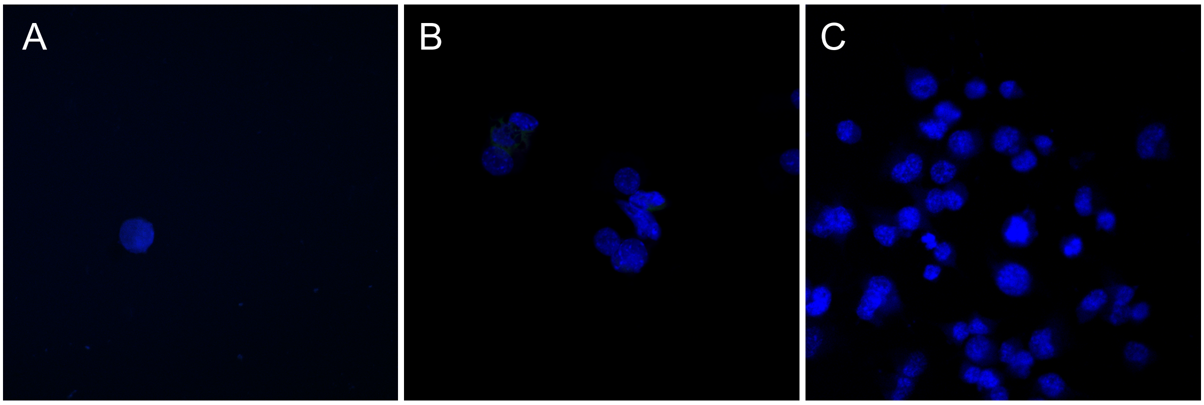

Fig. S3. Controls for immunocytochemistry experiments. *A,* laser scanning confocal microscopy image (maximum intensity projection) of a negative control for fluorescent *GLRA1* and *GLRB* constructs. Constructs with no fluorescent fused proteins were expressed for 48 hours followed by DAPI staining. No GlyR α_1 or β-subunit are detected. *B,* secondary antibody control excluding the primary anti-*GLRB* antibody. No green fluorescence is observed. *C,* immunostaining in the absence of transfected GlyR subunits as a control for primary antibody specificity. Scale bars, 10 µm.
